# Data regarding anti-quorum sensing and antimicrobial activity of *Melaleuca alternifolia* and *Salvia sclarea* essential oil against *Pseudomonas aeruginosa*

**DOI:** 10.1016/j.dib.2023.109145

**Published:** 2023-04-12

**Authors:** Anmol Srivastava, Vivek Kumar, Deepmala Sharma, Vishnu Agarwal

**Affiliations:** a Department of Biotechnology, Motilal Nehru National Institute of Technology, Allahabad (Prayagraj) 211004, Uttar Pradesh, India; b Department of Mathematics, National Institute of Technology Raipur, Raipur 492010, Chhattisgarh, India

**Keywords:** Pseudomonas aeruginosa, Quorum sensing, Virulence factors, Salvia sclarea, Melaleuca alternifolia, Essential oils

## Abstract

To combat the increasing number of multi-drug resistant bacteria, researchers are now looking for alternatives that reduce the virulence and pathogenic potential of the bacteria without killing it. It can be accomplished by interfering with the quorum sensing (QS) system of bacteria. In this article, we aim to determine the antimicrobial and anti-QS activity of *Salvia sclarea* and *Melaleuca alternifolia* essential oils (EOs) against *Pseudomonas aeruginosa*. The sub-lethal concentration of these EOs was found with the help of a growth curve, and further experiments were carried out below this concentration. To check for their anti-quorum activity, a bioreporter strain *E. coli* pJN105LpSC11 (to measure the concentration of 3-oxo-C_12__—_HSL) and *Chromobacterium violaceum* CV026 (to check for the reduction in the formation of violacein pigment) was used. Several virulence phenotype assays like pyocyanin, alginate, and protease production, along with swarming motility, were done. The effect of these EOs on biofilm formation was also checked. The results were confirmed by checking the expression of genes by real-time PCR.

## Graphical abstract

**Figure:**
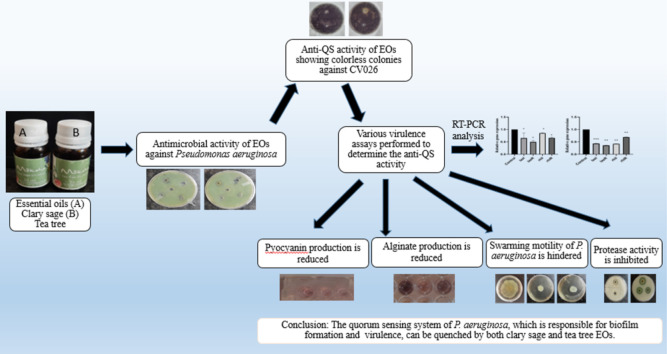

**Specifications Table**

**Table:** 

Subject	Biotechnology, microbiology, molecular biology
Specific subject area	Antimicrobial and Anti-quorum sensing activity of essential oils.
Type of data	Table, Figure (graphs and pictures)
How the data were acquired	Elisa plate reader was used to acquire data regarding the growth curve of *Pseudomonas aeruginosa* in presence of a different concentrations of essential oils. This instrument was also used to collect data from different experiments like virulence assays and biofilm formation. RT-PCR instrument from Applied Biosystems (StepOnePlus) was used to collect data regarding gene expression of *Pseudomonas aeruginosa* quorum sensing system.
Data format	Raw
Description of data collection	Both the *Melaleuca alternifolia* and *Salvia sclarea* EOs were purchased from Moksha Lifestyle products. Bacterial cultures were brought from MTCC and CTET, Spain, and grown in Luria-Bertani (LB) broth at the required temperatures. Dr. Peter Greenberg (University of Washington), USA, gave a reporter strain of *E. coli* pJN105LpSC11. We first checked the antibacterial activity and then explored the anti-Quorum sensing activity of these essential oils at sub-MIC concentration. RNA extraction from treated and control samples was done using the Total RNA Miniprep Purification Kit from HiMedia.
Data source location	Department of Biotechnology, Motilal Nehru National Institute of Technology Allahabad, Prayagraj, India·(25.4917° N, 81.8632° E)
Data accessibility	With the article and in repository Repository name: Mendeley Data Data identification number: 10.17632/94×7pjvxvw.2 Direct URL to data: https://data.mendeley.com/datasets/94×7pjvxvw/4
Related research article	Kalia M, Yadav VK, Singh PK, Sharma D, Pandey H, Narvi SS, Agarwal V. Effect of Cinnamon oil on quorum Sensing-Controlled Virulence Factors and Biofilm Formation in Pseudomonas aeruginosa. PLoS ONE. 10(8) (2015) e0135495. https://doi.org/10.1371/journal.pone.0135495

## Value of the Data

• In this paper, we are reporting the *Melaleuca alternifolia* and *Salvia sclarea* (EOs) for their antimicrobial and anti-quorum sensing activity.
• The gene expression of QS-related genes in the presence of *Melaleuca alternifolia* and *Salvia sclarea* EOs were also checked for the first time using RT-qPCR to validate the results. Hence, this information can be exploited to develop therapeutic medications to treat infections resulting from multi-drug resistant bacteria.
• This data can be beneficial to researchers working in the domain of quorum sensing and the genes involved in this regulation. It can also assist in studies relating to virulence and *Pseudomonas aeruginosa*.

## 1 Objective

The National Institute of Health (NIH) reports that almost 65% of microbial infections are related to the formation of biofilm. Due to the excessive use of antibiotics, the number of multi-drug resistant bacteria is increasing. So, to overcome this problem, newer strategies are being adopted that exert low selection pressure on the bacteria. This study aims to examine the possibility of *Salvia sclarea* (common name is Clary sage) and *Melaleuca alternifolia* (common name is Tea tree) EOs to show antimicrobial and anti-quorum sensing activity against *Pseudomonas aeruginosa*, which is responsible for a large percentage of nosocomial infections and diseases in immuno-compromised patients due to the formation of biofilm. *Salvia sclarea* essential oil (EO) has been known for its anti-inflammatory, antiviral, antibacterial, and antimalarial properties. On the other hand, *Melaleuca alternifolia* EO has been reported to have antiprotozoal activity in addition to antibacterial, antifungal, and antiviral properties.

## 2 Data Description

**Fig. 1:**
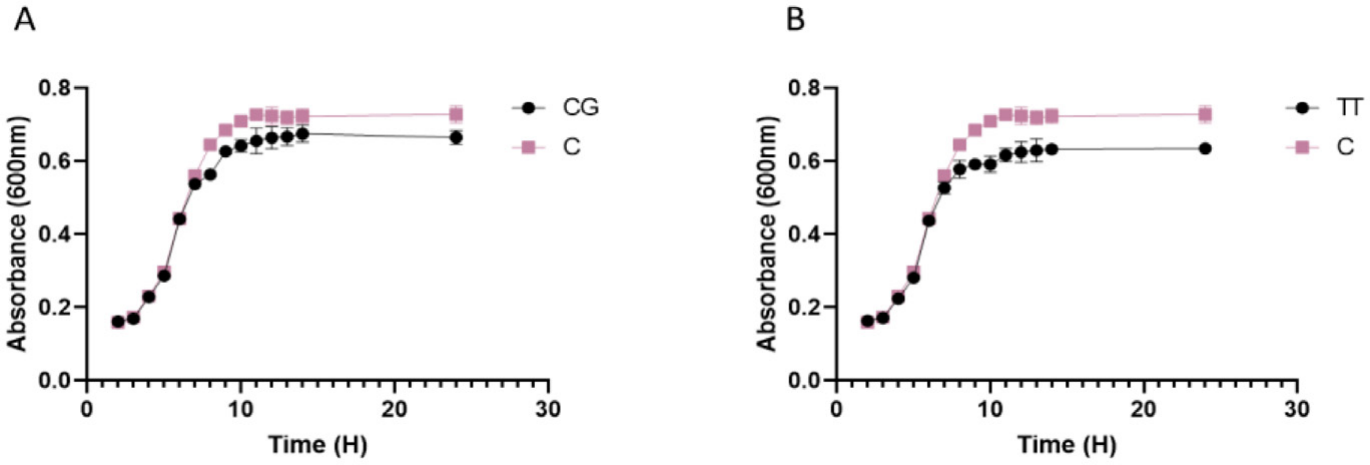
Growth curve of *P. aeruginosa* in the presence and absence of sub-lethal concentration (5 µl/ml for CG and 1.25 µl/ml for TT) of EO (A) *Salvia sclarea* (CG) and (B) *Melaleuca alternifolia* (TT) EO (C) Control (C). All the experiments of this study were performed three times, and the error bars represent the mean standard error of three readings.

**Fig. 2:**
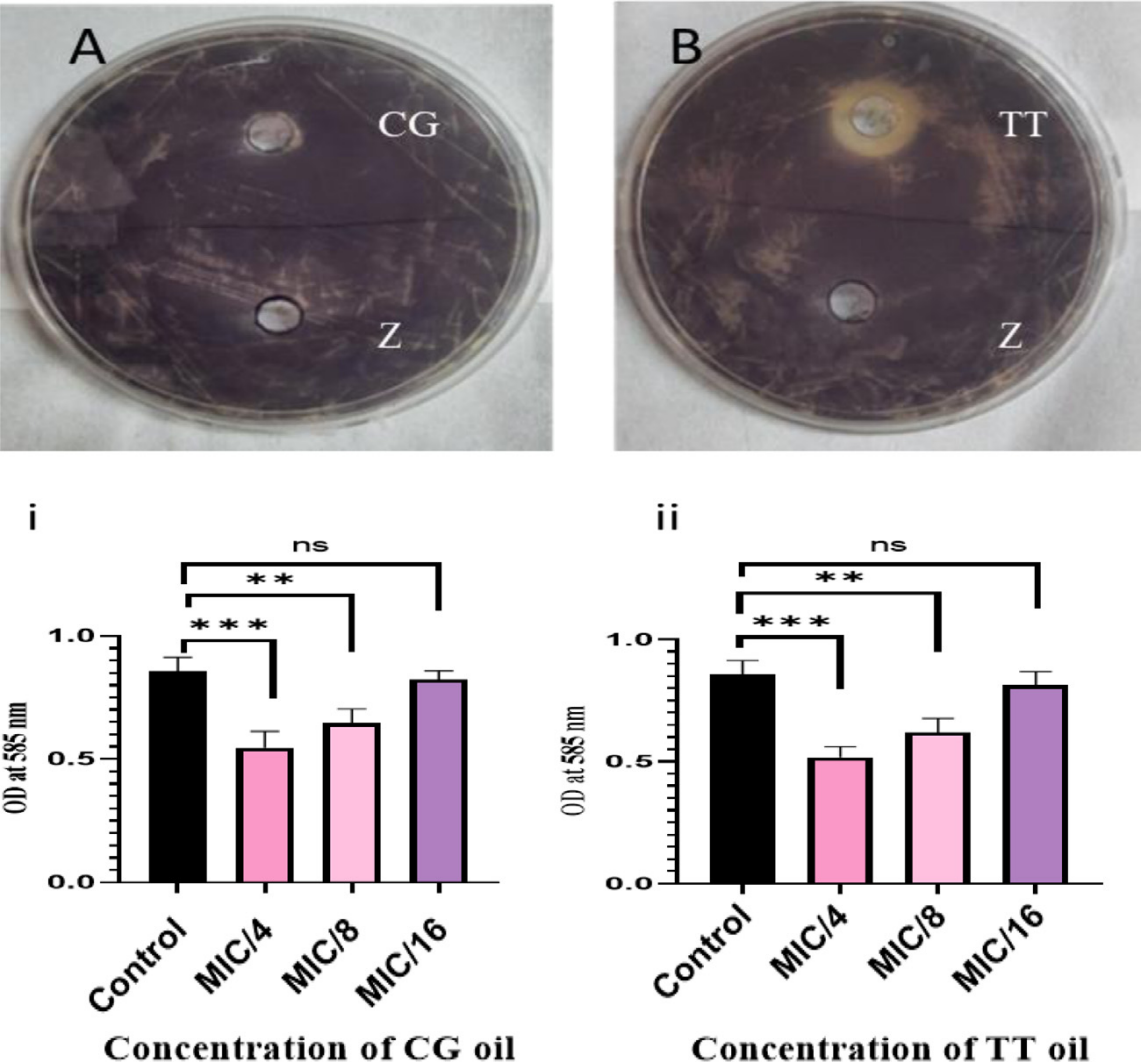
Anti-QS activity of EO against *Chromobacter violaceum* CV026; Halo zone formation (representing colorless colonies) (A) well containing of *Salvia sclarea* (CG) EO (B) well containing *Melaleuca alternifolia* (TT) EO (Z) well containing media along with DMSO (control), but no EO. (i) Quantitative inhibition of violacein pigment in the case of CG EO (ii) quantitative estimation of violacein pigment production in the case of TT EO. Control used was media along with DMSO and untreated *Chromobacterium violaceum* CV026 bacterial culture. ns represents not significant. Here the concentration of essential oils used for the assay is mentioned in the form of MIC fractions where MIC/4 represents quarter strength of the MIC determined, MIC/8 represents one eighth of the MIC calculated and MIC/16 represents one sixteenth of the MIC calculated. All the experiments of this study were performed three times, and the error bars represent the mean standard error of three readings. ** *P*< 0.001 in comparison to control. *** *P*< 0.0001 in comparison to control.

**Fig. 3:**
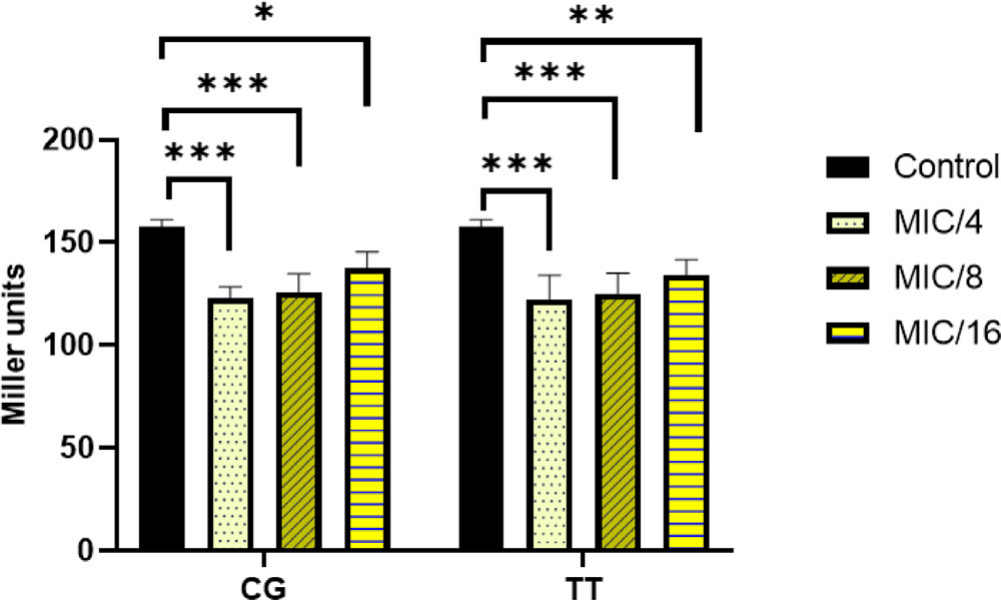
Effect of EOs on the concentration 3-oxo-C_12__—_HSL produced by *P. aeruginosa* PA01, measured by β-Galactosidase activity. Control used was DMSO treated culture of *P. aeruginosa* PA01. Here the concentration of essential oils used for the assay is mentioned in the form of MIC fractions where MIC/4 represents quarter strength of the MIC determined, MIC/8 represents one eighth of the MIC calculated and MIC/16 represents one sixteenth of the MIC calculated. All the experiments of this study were performed three times, and the error bars represent the mean standard error of three readings * *P*< 0.05 compared with the control. ** *P*< 0.001 in comparison to control. *** *P*< 0.0001 in comparison to control.

**Fig. 4:**
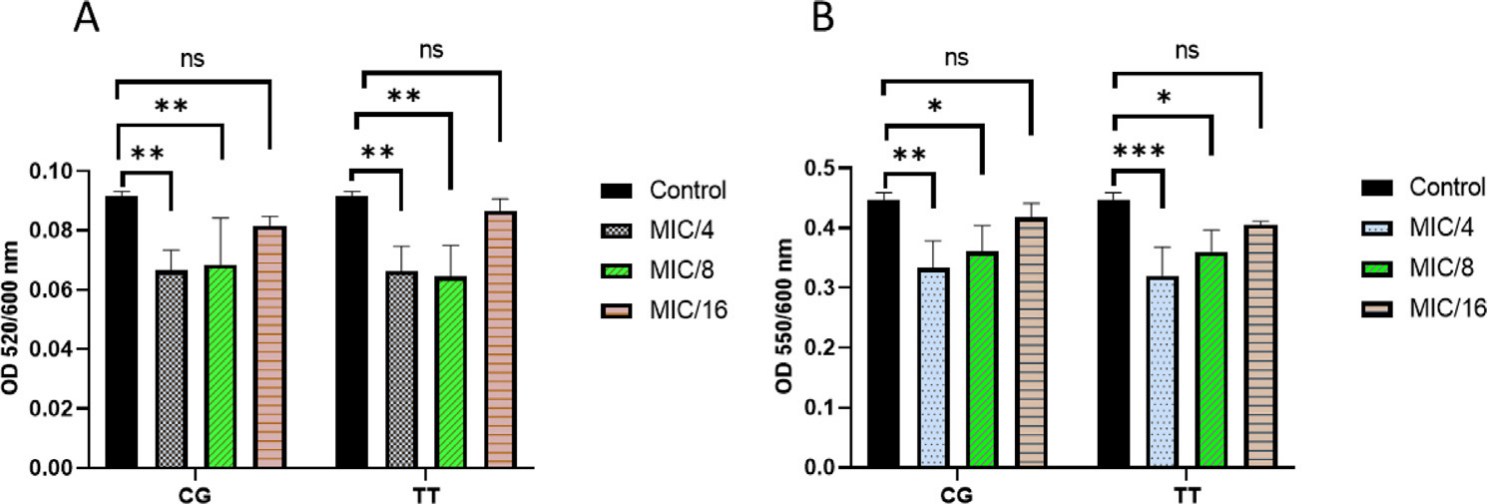
Effect of EOs on virulence factors of *P. aeruginosa* PA01 (A) Inhibition in the production of pyocyanin by EOs at sub-lethal concentration of EOs. Normalization of data was done according to OD_600nm_. (B) Effect of EO on alginate production by PA01. Control used was DMSO treated culture of *P. aeruginosa* PA01 ns represents not significant. Here the concentration of essential oils used for the assay is mentioned in the form of MIC fractions where MIC/4 represents quarter strength of the MIC determined, MIC/8 represents one eighth of the MIC calculated and MIC/16 represents one sixteenth of the MIC calculated. All the experiments of this study were performed three times, and the error bars represent the mean standard error of three readings* *P*< 0.05 compared with the control. ** *P*< 0.001 in comparison to control. *** *P*< 0.0001 in comparison to control.

**Fig. 5:**
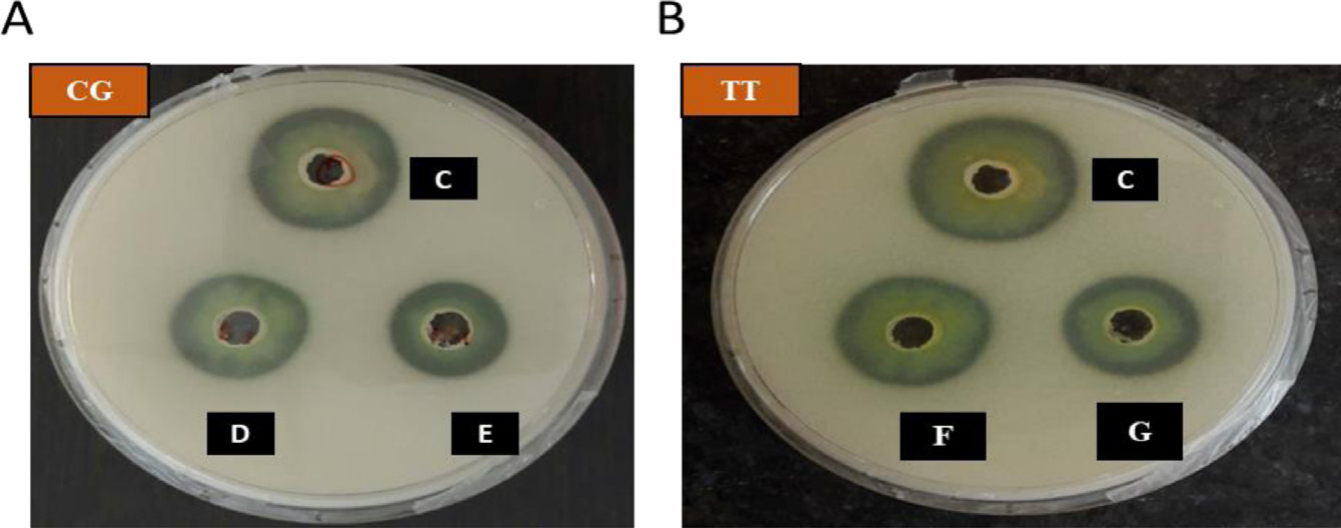
Inhibition of protease production carried out using skim milk agar assay (A) In the presence of *Salvia sclarea* oil (C-control, d-MIC/8, E-MIC/4) (B) In the presence of *Melaleuca alternifolia* oil (C-control, F-MIC/8, G-MIC/4). Control used was DMSO treated culture supernatant of *Pseudomonas aeruginosa* PA01.

**Fig. 6:**
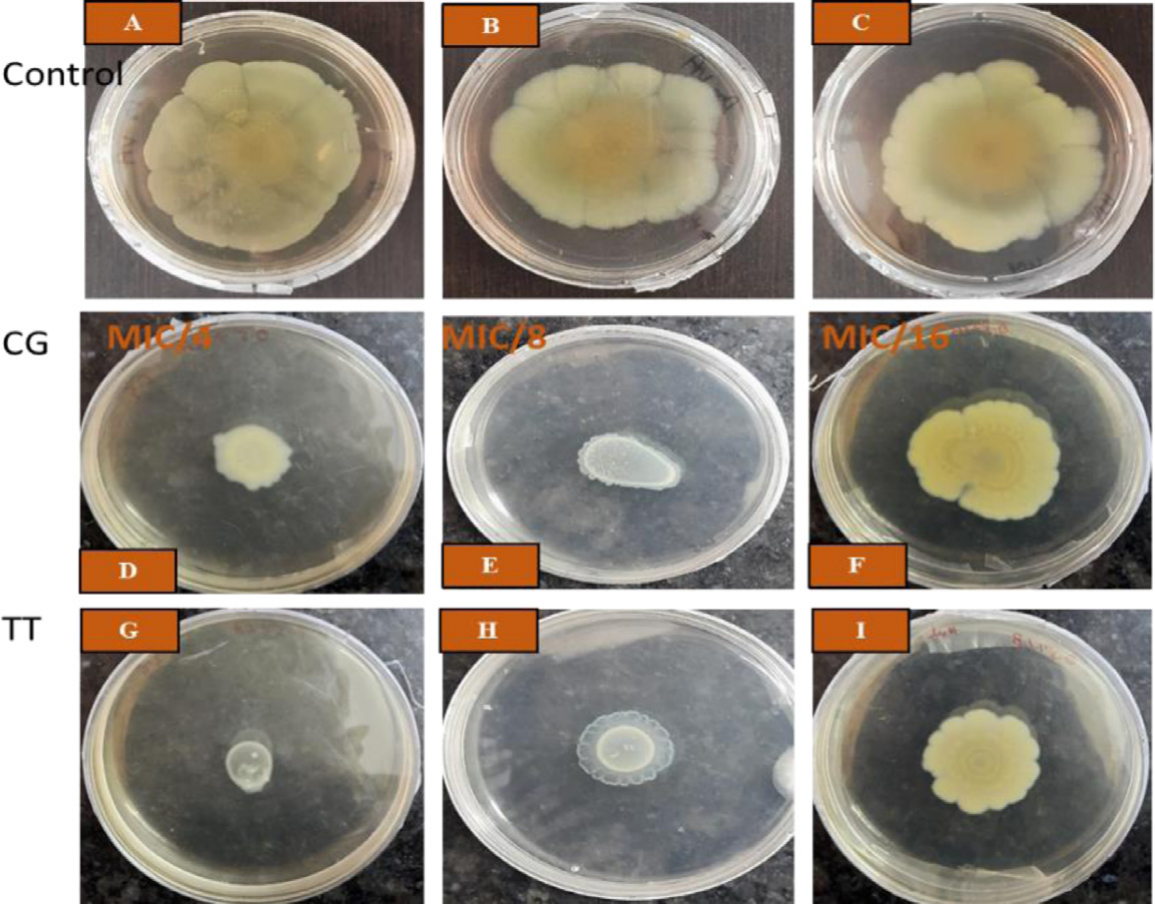
Swarming motility of *P. aeruginosa* PA01 in swarm agar media (A (untreated PA01), B (PA01 in the presence of DMSO), C (untreated PA01)) control (absence of EO), (D, E, F) in the presence of *Salvia sclarea* EO (G, H, I) in the presence of *Melaleuca alternifolia* EO. Here the concentration of essential oils used for the assay is mentioned in the form of MIC fractions where MIC/4 represents quarter strength of the MIC determined, MIC/8 represents one eighth of the MIC calculated and MIC/16 represents one sixteenth of the MIC calculated.

**Fig. 7:**
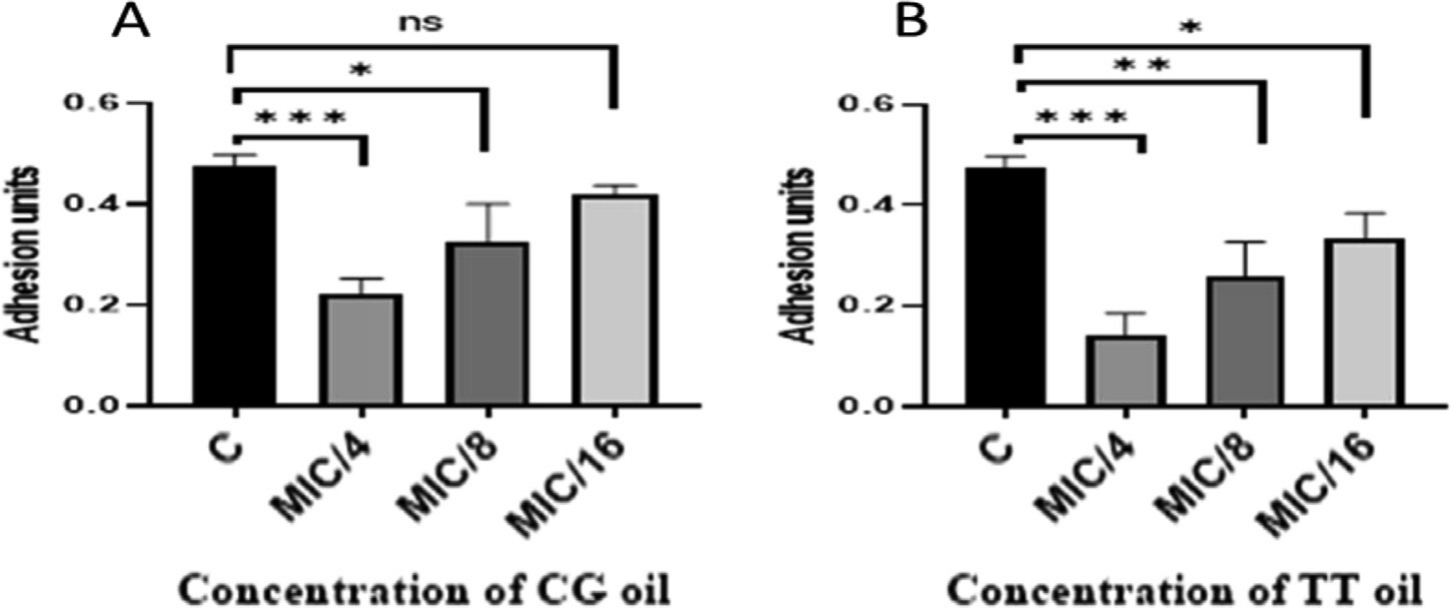
Inhibition in biofilm formation at different concentrations below sub-lethal concentration is represented in the form of adhesion units i.e., OD_590nm_/OD_600nm_ (A) in the presence of decreasing concentration of *Salvia sclarea* EO (B) in the presence of decreasing concentration of *Melaleuca alternifolia* EO. Control used was DMSO treated culture of *P. aeruginosa* PA01. ns represents not significant. Here the concentration of essential oils used for the assay is mentioned in the form of MIC fractions where MIC/4 represents quarter strength of the MIC determined, MIC/8 represents one eighth of the MIC calculated and MIC/16 represents one sixteenth of the MIC calculated. The experiments were repeated thrice and the error bars indicate standard error mean of three readings. * *P*< 0.05 compared with the control. ** *P*< 0.001 in comparison to control. *** *P*< 0.0001 compared with the control.

**Fig. 8:**
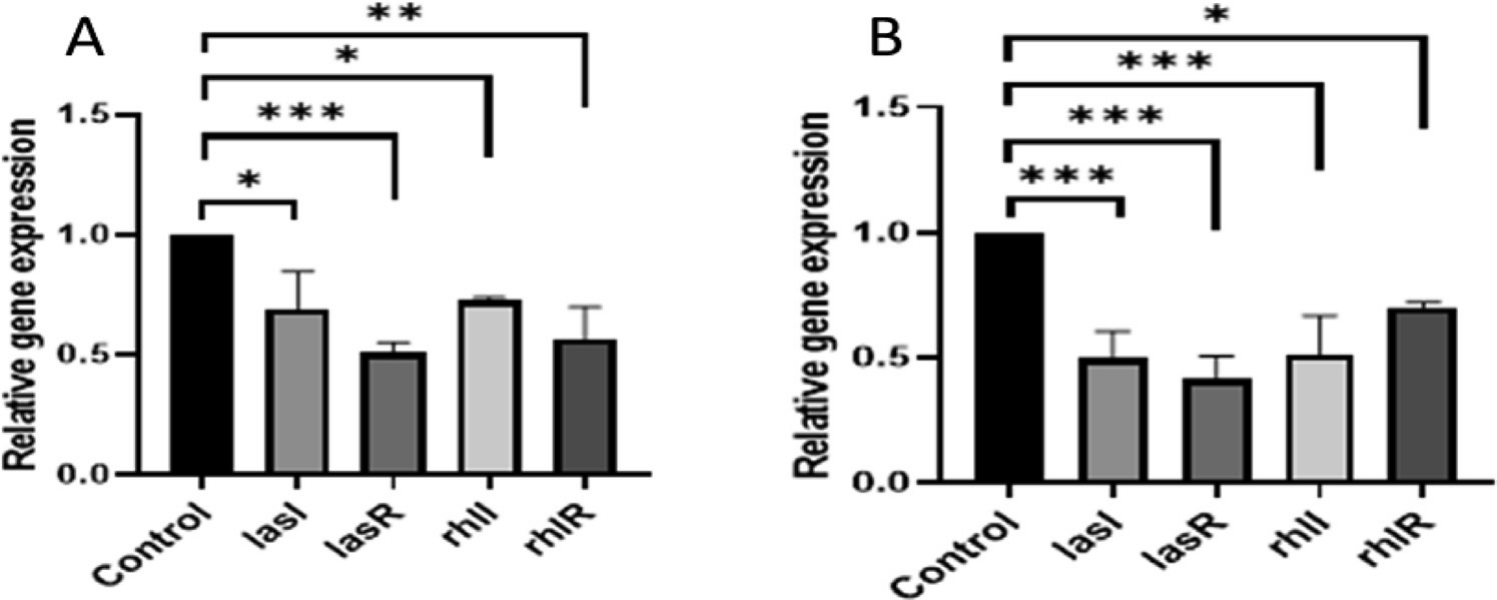
Gene expression analysis of QS regulatory genes of *Psedudomonas aeruginosa* PA01 by RT-PCR in the presence of MIC/4 concentration of EO (A) *Salvia sclarea* EO (B) *Melaleuca alternifolia* EO. The experiments were repeated thrice and the error bars indicate standard error mean of three readings. Control used was DMSO treated culture of *P. aeruginosa* PA01 * *P*< 0.05 in comparison to control. ** *P*< 0.001 compared with control. *** *P*< 0.0001 compared with control.

Herbal extracts and essential oils are gaining worldwide attention for their therapeutic properties against a wide species of bacteria. In this regard, we explored the antibacterial and ant-QS activity of *Melaleuca alternifolia* and *Salvia sclarea* EOs against a multi-drug resistant bacteria *Pseudomonas aeruginosa* PA01. The determination of MIC of EOs was done using a 96-well microtiter plate with each well having different concentrations ofEOs ranging from 80 µl/ml to 0.125 µl/ml. The MIC calculated for these EOs is mentioned in Table 1. The growth curve was plotted at different concentrations; however, only that concentration of EO was selected for further experiments that did not significantly affect the growth kinetics (displayed in Fig. 1) of *P. aeruginosa* and mimicked the growth pattern of the control. The anti-QS inhibition activity of *Salvia sclarea* and *Melaleuca alternifolia*, EO against *Pseudomonas aeruginosa* was assessed using CV026 biosensor strain as depicted in Fig. 2. DMSO was used as a control here. β-Galactosidase assay was performed to determine the effect of EO on the production of 3-oxo-C_12__—_HSL by *P. aeruginosa* as presented in Fig. 3. Here, the control was an untreated PA01 culture, and the result was expres sed in the form of miller units. To check for the effect of EOs on the virulence phenotypes under the control of the QS system, several assays were performed like pyocyanin Fig. 4(A). The effect of *Salvia sclarea* and *Melaleuca alternifolia* EOs on the alginate production by *Pseudomonas aeruginosa* is represented in Fig. 4(B). To estimate the level of protease activity in the treated and untreated culture of *P. aeruginosa*, protease assay was performed as presented in Fig. 5 and the data regarding the zone of protease inhibition is mentioned in Table 2. Swarming motility of the treated culture and control (untreated culture) was assessed and the result is presented in Fig. 6 and Table 3*.* The data regarding potential of EOs at different concentrations to inhibit biofilm formation is given in Fig. 7. Fig. 8 depicts the gene expression studies, which reflect the effect of EOs on quorum sensing (QS) system genes of *P. aeruginosa* like *lasI, lasR, rhlI, rhl*.

**Table 1:** Minimum inhibitory concentration (MIC) and concentration of essential oils that doesn't affect the growth kinetics of *Pseudomonas aeruginosa* PA01. Growth mimicking concentration of essential oils is reported in terms of MIC fractions where MIC/4 represents quarter strength of the MIC determined.

Essential oil	MIC (µl/ml)	Growth mimicking concentration of essential oils (µl/ml)
*Salvia sclarea* (Clary sage)	20 µl/ml	5 µl/ml (MIC/4)
*Melaleuca alternifolia* (Tea tree)	5 µl/ml	1.25 µl/ml (MIC/4)

**Table 2:** Zone of protease inhibition in mm (in skim milk agar plate), here the concentration of essential oils used for the assay is mentioned in the form of MIC fractions where MIC/4 represents quarter strength of the MIC determined and MIC/8 represents one eighth of the MIC calculated.

Essential oil	Zone of protease inhibition (mm)		
*Salvia sclarea* (CG)	MIC/4	MIC/8	Control (DMSO treated PA01)
	12	15	18
*Melaleuca alternifolia* (TT)	14	17	21

**Table 3:** Swarming motility of *Pseudomonas aeruginosa* PA01 in the presence of sub-MIC concentration of essential oils, Here the concentration of essential oils used for the assay is mentioned in the form of MIC fractions where MIC/4 represents quarter strength of the MIC determined, MIC/8 represents one eighth of the MIC calculated and MIC/16 represents one sixteenth of the MIC calculated.

Essential oil	Swarming motility (mm)		
*Salvia sclarea*	MIC/4	MIC/8	MIC/16
	11	13	26
*Melaleuca alternifolia*	8	14	21
Control	63 (untreated PA01)	68 (DMSO treated PA01)	66 (untreated PA01)

## 3 Experimental Design, Materials and Methods

### 3.1 Essential Oils

Both the *Melaleuca alternifolia* and *Salvia sclarea* EOs were purchased from Moksha Lifestyle products. The manufacturer provided its certificate of analysis, and GC–MS report containing the list of chemical compounds. Being hydrophobic, they were dissolved in DMSO, purchased from SRL, India.

### 3.2 Microorganisms and Culture Conditions

*Pseudomonas aeruginosa* PA01 was purchased from the MTCC. *Chromobacterium violaceum* CV026, a mini-Tn5 mutant (ATCC31352), was purchased from CTET, Spain, and was grown in Luria-Bertani (LB) broth at 28°C broth in the presence of kanamycin (25 µg/ml). N-hexanoyl-homoserine lactone (HHL) was added to the CV026 culture when we needed to screen EOs for anti-QS activity. It was purchased from Sigma-Aldrich. Dr. Peter Greenberg (University of Washington), USA, gave a reporter strain of *E. coli* pJN105LpSC11 that was gentamycinand ampicillin-resistant. The concentration of gentamycin and ampicillin that was used to culture *E. coli* pJN105LpSC11 was 10 µg/ml and 100 µg/ml respectively. All these bacterial cultures were grown overnight in an incubator at 120 rpm.

### 3.3 Antimicrobial Activity

The antimicrobial activity of EOs was tested against *Pseudomonas aeruginosa* PA01. Briefly, the bacteria were grown overnight at 37°C, 120 rpm and used for the experiment when its OD reached 0.5 at 600 nm. Then the culture was spread uniformly on the LBA plates, and the wells were punched into which 100 µl of different concentrations of EOs was pipetted. Then the plates were kept in an incubator for overnight at 37°C. The antimicrobial potential of both *Salvia sclarea* and *Melaleuca alternifolia* EOs was measured by the diameter of the zone of inhibition (ZOI) around the well.

### 3.4 Calculation Of Minimum Inhibitory Concentration (MIC) of EOs

Micro-broth dilution method was used to determine the MIC of *Salvia sclarea* and *Melaleuca alternifolia* EO against PA01 [1]. The culture was grown overnight, which, when it reached an OD of 1 at 600 nm, was mixed with new media in a 96-well plate, and allowed to grow in the presence of different concentrations of EOs. The plate was incubated overnight at 37°C, and the minimum concentration that inhibited the growth of bacteria completely was taken as the MIC of that EO.

### 3.5 Growth Curve Analysis of EOs

It was done to find the sub-lethal concentration of EOs. In brief, the overnight grown PA01 culture (OD_600nm_ = 1) was inoculated into fresh media containing different concentrations of EOs for 24 h; the plate was kept in an incubator at 37°C, and OD was taken regularly at an interval of 2 h.

### 3.6 Qualitative Screening for QS Inhibition by Essential Oils

The anti-QS activity of EOs was determined by a bioreporter strain, *Chromobacter violaceum* CV026. Briefly, LB agar plates were made that contain hexanoyl homoserine lactone (0.125 µg/ml) and antibiotic kanamycin (20 µg/ml), and 100 µl of an overnight grown culture of *C. violaceum* was spread on the plate uniformly, and 100 µl of EO was added into the wells, and the plates were kept overnight at 28°C and observed for colorless colonies [2].

### 3.7 Quantitative Estimation of 3-oxo-C_12_—HSL

To assess the effect of EOs on the level of autoinducer 3-oxo-C_12__—_HSL, reporter strain *E coli* DH5α pJN105LpSC11 was cultured overnight in LB media containing ampicillin and gentamycin. Then this culture was inoculated into fresh LB media, and at OD_600nm_ 0.2, LasR expression was induced by 0.2% (w/v) l-arabinose and left for incubation until the OD_600nm_ reached 0.5. The treated culture was centrifuged for 10 min at 10,000 rpm, and the supernatant was filtered and mixed with 1:1000 dilution of *E coli* DH5α pJN105LpSC11 and kept for incubation at 37°C for 2 h. The concentration of 3-oxo-C_12_—HSL was then determined by miller assay [3,4].

### 3.8 Effect of EOs on Virulence Phenotypes

#### 3.8.1 Pyocyanin Production

PA01 culture was grown in the presence of EOs individually. The culture was then centrifuged, and the supernatant was separated and filtered. Chloroform (1:1 vol) was used to extract pyocyanin and, the brownish upper part was discarded, the blue-colored pyocyanin pigment was visible, which was further extracted by 0.2 N HCL, and yielded pink color. The OD was measured at 520 nm [5].

#### 3.8.2 Protease Assay

Protease production by *P. aeruginosa* was estimated by skim milk agar assay. The PA01 culture was treated overnight with EOs and then centrifuged at 10,000 rpm for 10 min; the supernatant was then filter sterilized. Next, 100 µl of this supernatant was added to wells cut on a skim milk agar plate. This plate was then incubated overnight at 37°C, and the zone of clearance around each well was observed [6].

#### 3.8.3 Swarming Motility Assay

Briefly, swarm agar plates containing yeast extract, bacto agar (0.4% (w/v)), bacto peptone, and glucose were made and its pH was maintained at 7.0. *P. aeruginosa* culture was treated overnight in the presence of EOs. 10 µl of overnight culture was pipetted onto the middle of the swarm plate, and these plates were kept in an incubator at 37°C for overnight [7].

#### 3.8.4 Alginate Assay

PA01 culture was grown overnight and inoculated in fresh media in the presence and absence of EOs, 500 µl of 1 M NaCl was mixed with an equal volume of treated culture and subjected to centrifugation. Then, 500 µl of 0.1% (w/v) cetylpyridinium chloride (CPC) was mixed into the supernatant, and the tubes were centrifuged at 10,000 rpm for 10 min at room temperature. Then 500 µl chilled isopropanol was used to re-suspend the pellet. After that, the tube was centrifuged again, and the supernatant was discarded; then, 1 M NaCl was added to the tube to re-suspend the pellet, which was left overnight. Carbazole assay was further carried out to estimate the alginate concentration in culture [8]. Briefly, 50 µl of sample was added to 200 µl of 25 µM sodium tetraborate (in H_2_SO_4_), the plate was then heated for 10 min at 100°C in an oven. After cooling at room temperature, we added 50 µl of 0.1% carbazole (in absolute ethanol) to it and then again heated the plate for 15 min at 100°C. The final absorbance was then taken at 550 nm.

### 3.9 Inhibition of Biofilm Formation by EOs

*P. aeruginosa* PA01 culture was adjusted to OD_600nm_=1 and then inoculated into fresh LB media, and 100 µl of this culture was poured into individual wells of a 96-well microtiter plate. Then the sub-lethal concentration of EOs was mixed with the culture, and the plate was kept overnight at 37°C. Fresh LB media was prepared and poured into a new 96-well plate, and 1 µl of culture was dispensed into wells. The culture in the wells was pipetted out, and phosphate buffer saline (PBS) was used to wash the wells. Next, the wells were stained for 20 min with crystal violet dye (0.4% (w/v)). The dye was discarded, and the wells were rinsed thrice with distilled water till all the unbound dye was removed from the wells. Next, the stain was solubilized by adding 100 µl DMSO, and the optical density (OD) of the wells was taken at 590 nm and 600 nm [9,10].

### 3.10 Gene Expression Analysis Through RT-QPCR

In brief, the bacterial culture was treated with EOs, and mRNA was extracted from it, which was further used to synthesize cDNA using RevertAid First Strand cDNA synthesis kit. The reaction mixture included 5 µl SYBR green, 500 ng cDNA, 10 µM forward and reverse primers, and 2 µl distilled water. The plate was then subjected to real-time PCR analysis, and the reaction was completed in 2 steps; the first step is initial denaturation for 30 s, while the second step included denaturation for 60 s and annealing for 30 s at 60°C; the cycle was repeated 40 times. The results obtained from RT-PCR were in the form of C_t_ values, which were used to calculate fold change with the help of ∆∆C_t_ method. The result was reported in the form of relative gene expression [11].

### 3.11 Statistical Analysis

All the experiments in this study were conducted thrice, and the results are expressed in terms of average values and their standard mean error. The significance of the values was checked by one-way analysis of variance (ANOVA) using GraphPadPrism, and only values at *p*<0.05 were taken to be significant.

## Ethics Statements

This work does not involve any human subject or any trial on animal. Social media was not used to collect any data. This data article adheres to all the ethics necessary for publishing.

## CRediT authorship contribution statement

**Anmol Srivastava:** Data curation, Writing – original draft, Visualization, Investigation. **Vivek Kumar:** Supervision. **Deepmala Sharma:** Software, Validation. **Vishnu Agarwal:** Conceptualization, Methodology, Software, Writing – review & editing.

## Declaration of Competing Interest

The authors declare that they have no known competing financial interests or personal relationships that could have appeared to influence the work reported in this paper.

## Data Availability

Data regarding anti-quorum sensing and antimicrobial activity of Melaleuca alternifolia and Salvia sclarea essential oil against Pseudomonas aeruginosa (Original data) (Mendeley Data).
